# Integrating row level security in i2b2: segregation of medical records into data marts without data replication and synchronization

**DOI:** 10.1093/jamiaopen/ooad068

**Published:** 2023-08-14

**Authors:** Raphael Scheible, Fabian Thomczyk, Marco Blum, Micha Rautenberg, Andrea Prunotto, Suhail Yazijy, Martin Boeker

**Affiliations:** Institute of Artificial Intelligence and Informatics in Medicine (AIIM), Chair of Medical Informatics, University Hospital rechts der Isar, School of Medicine, Technical University of Munich, Munich, Germany; Center for Chronic Immunodeficiency (CCI), Medical Center, Faculty of Medicine, University of Freiburg, Freiburg, Germany; Data Inintegration Center (DIC), University of Freiburg, Freiburg, Germany; Data Inintegration Center (DIC), University of Freiburg, Freiburg, Germany; Institute of Medical Biometry and Statistics, Medical Center, Faculty of Medicine, University of Freiburg, Freiburg, Germany; Zentrum für Digitalisierung und Informationstechnologie (ZDI), Medical Center, University of Freiburg, Freiburg, Germany; Data Inintegration Center (DIC), University of Freiburg, Freiburg, Germany; Institute of Medical Biometry and Statistics, Medical Center, Faculty of Medicine, University of Freiburg, Freiburg, Germany; Institute of Artificial Intelligence and Informatics in Medicine (AIIM), Chair of Medical Informatics, University Hospital rechts der Isar, School of Medicine, Technical University of Munich, Munich, Germany

**Keywords:** i2b2, databases, data-warehousing, data segregation, data security, software

## Abstract

**Objective:**

i2b2 offers the possibility to store biomedical data of different projects in subject oriented data marts of the data warehouse, which potentially requires data replication between different projects and also data synchronization in case of data changes. We present an approach that can save this effort and assess its query performance in a case study that reflects real-world scenarios.

**Material and Methods:**

For data segregation, we used PostgreSQL’s row level security (RLS) feature, the unit test framework pgTAP for validation and testing as well as the i2b2 application. No change of the i2b2 code was required. Instead, to leverage orchestration and deployment, we additionally implemented a command line interface (CLI). We evaluated performance using 3 different queries generated by i2b2, which we performed on an enlarged Harvard demo dataset.

**Results:**

We introduce the open source Python CLI i2b2rls, which orchestrates and manages security roles to implement data marts so that they do not need to be replicated and synchronized as different i2b2 projects. Our evaluation showed that our approach is on average 3.55 and on median 2.71 times slower compared to classic i2b2 data marts, but has more flexibility and easier setup.

**Conclusion:**

The RLS-based approach is particularly useful in a scenario with many projects, where data is constantly updated, user and group requirements change frequently or complex user authorization requirements have to be defined. The approach applies to both the i2b2 interface and direct database access.

## Background and significance

Today, data warehouses form the foundation of modern data science in all areas where insights are gained from data. However, Big Data requires intelligent solutions to ensure privacy protection through controlled access to data; especially in the medical and healthcare sector, data access should only be possible for authorized persons. In addition, fine-grained access control concepts are required to map access to different subsets of data in a warehouse according to the rights of users.^1–3^

Within the medical field i2b2 is an established open source data warehouse software that is used for many types of applications. The platform comes with a web application featuring a user interface, which is primarily designed for cohort identification based on parameterizable queries. Therefore, the i2b2 database is realized using a special entity-attribute-value model,^4^^,^^5^ which is very flexible and allows applications and extensions of i2b2.^6–8^ This architecture can be utilized for generic patient data storage. Consequently, i2b2 has been used in a variety of medical applications such as an integrated data repository for cancer research or as a clinical data repository.^6^^,^^9^ When using i2b2 to supply groups of users to restricted cohorts of the complete data warehouse, usually replication and synchronization of data has been necessary between projects. I2b2 realizes these projects by data marts, conceivable as mini databases, offering the possibility for specific data segregation. The core components of i2b2 itself are separated into multiple independent web services, called Cells. The Cell responsible for handling data marts is called the Data Repository Clinical Research Chart Cell. Each Clinical Research Chart (CRC) is linked to its own database server, instance or schema. This decentralized concept allows the replication of data subsets into data marts, while sharing other components from a central instance. Access to clinical data via the web interface is managed through i2b2 projects, users, and project roles, which are defined in the Project Management (PM) Cell. A Data Repository can be registered for multiple projects. A user can have multiple roles in multiple projects, giving further options on access control. This enables the representation of structures and hierarchies as found in many clinical and scientific institutions. However, depending on data size, as well as the variety and complexity of required access rights, the data mart approach will most likely lead to data replication and effort to keep all storage in sync. i2b2 supports PostgreSQL as a database management system, which obtained a significant improvement of security capabilities with the release of version 9.5 in 2016: Row level security (RLS). RLS introduced the design of user permission policies that can restrict access horizontally to only defined rows of a relational database table. Until then the original privileges management system existed, only providing features to vertically restrict user access on columns of tables and views. Consequently, RLS in combination with the default privileges system of PostgreSQL offers many possibilities to restrict data access for specific users and operations on a database level.^10^^,^^11^ Further, PostgreSQL 10 significantly improved the performance and scalability of RLS.^12^ To our knowledge, a user access management system for i2b2 based on RLS has not been described so far. Addressing this shortcoming with the capabilities of PostgreSQL we present an approach that realizes the functionality of data marts leveraged by RLS and directly tackles the i2b2’s google group discussion asking for a way to easily combine 2 i2b2 projects.^13^

## Objectives

The aim of this work is to develop an approach for the realization of data segregation similar to data marts using Role Level Security (RLS). Additionally, the functionality, correctness and performance of the presented solution is evaluated in comparison to the classical approach in a case study.

## Materials and methods

### Data segregation approach

In this article, we propose a data segregation approach that supports centralized management and is implemented without modifying the i2b2 code, but using the built-in technology and its features. The core of the proposed data segregation approach is RLS, which we use to define specific access rules associated with security roles. In our case, some use cases require that user access is restricted to self-provisioned data or data from a specific department. In addition, some tables may require global read-only access, except for data entered by the user. RLS is a native Postgres feature that allows easy implementation of such features by creating policies in SQL. The policies can be customized at any time and the access restriction applies immediately after the DBMS successfully processed the SQL code.

Since errors in access policies are critical, test-driven development based on the Test Anything Protocol (TAP)^14^ is a key feature of our approach. For Postgres, there exists an extra version called pgTAP^15^ that is integrated into the Postgres Docker container of our i2b2 stack.

The tests are implemented with their security role constraints and must pass successfully. In our case, if a test fails, the changes already made before the failed test are rolled back so that the failed security role is not deployed. In the long run, the risk of implementation failures is reduced.

**Figure 1.**
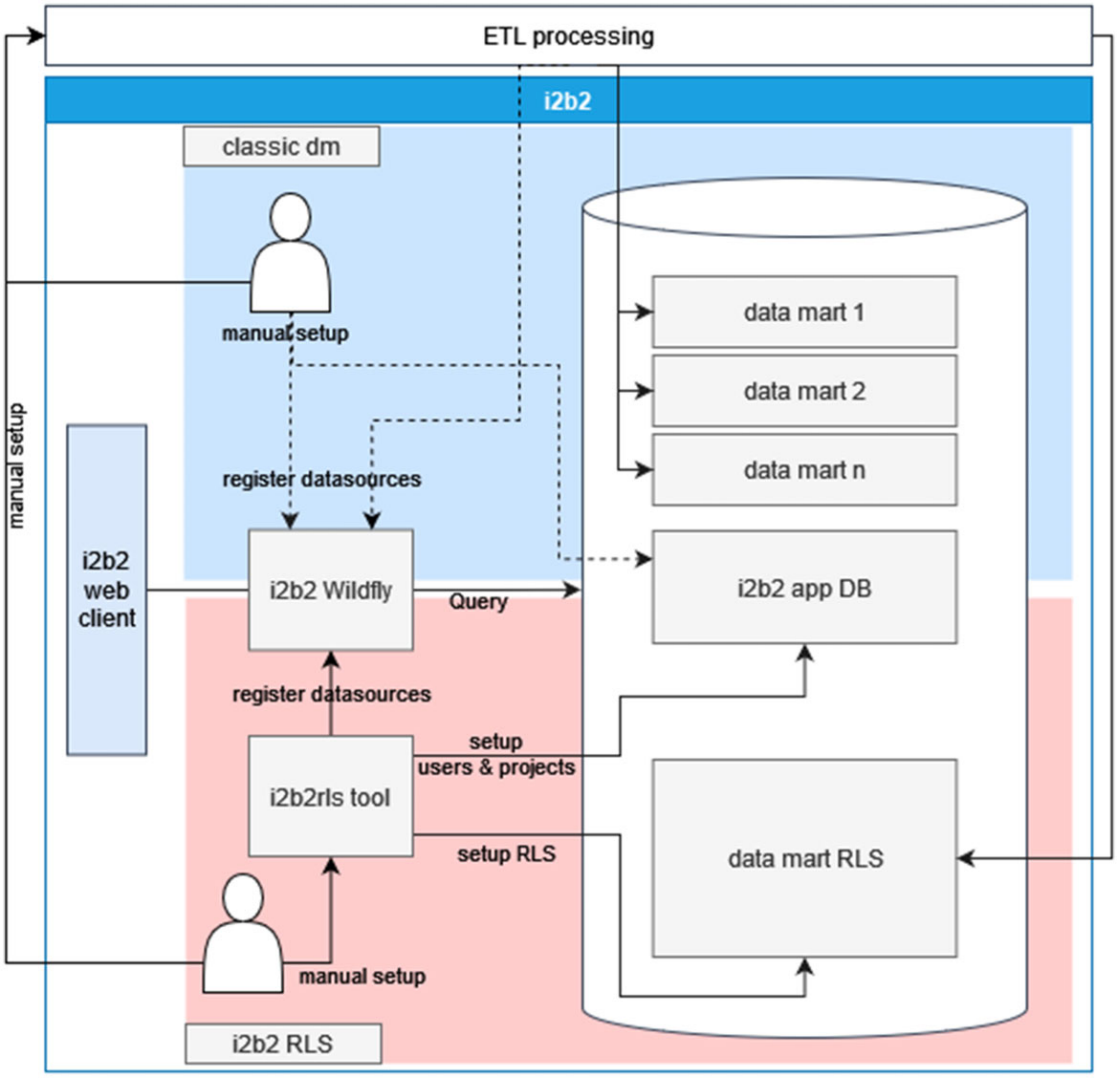
This schema provides an overview of the i2b2 stack components and the approach of the i2b2rls tool to manage required resources in the stack and compares it to the classic data mart creation. With the classic data mart one has to setup i2b2 components manually or incorporate these manipulations into the ETL process, which creates the data marts by potential data replication. With the i2b2rls tool one has to create the tool's setup, which might include creating new access policies by hand. However, the complexity of the ETL process potentially shrinks and there is no replication involved. Alternatively, one could also setup multiple i2b2 instances, which involves several additional tasks.

To conveniently orchestrate and deploy the entire i2b2 projects and data marts implemented with RLS, the command line interface (CLI) i2b2rls was developed. I2b2 cells, users, projects, project roles, and other configuration options are defined in a configuration file that is read when the CLI is started. Security roles are defined in SQL files and organized in a predefined structure. i2b2rls can be used to create, read, and delete the resources required to register a new i2b2 project and run the corresponding SQL code for a project's data source. Figure 1 illustrates the components with which the tool works.

The CLI i2b2rls was developed using the Python programming language and relies heavily on the AutoMapDB framework,^16^ whereas all the presented work is based on PostgreSQL 13 and i2b2 1.7.12a.

### Case study design

**Figure 2.**
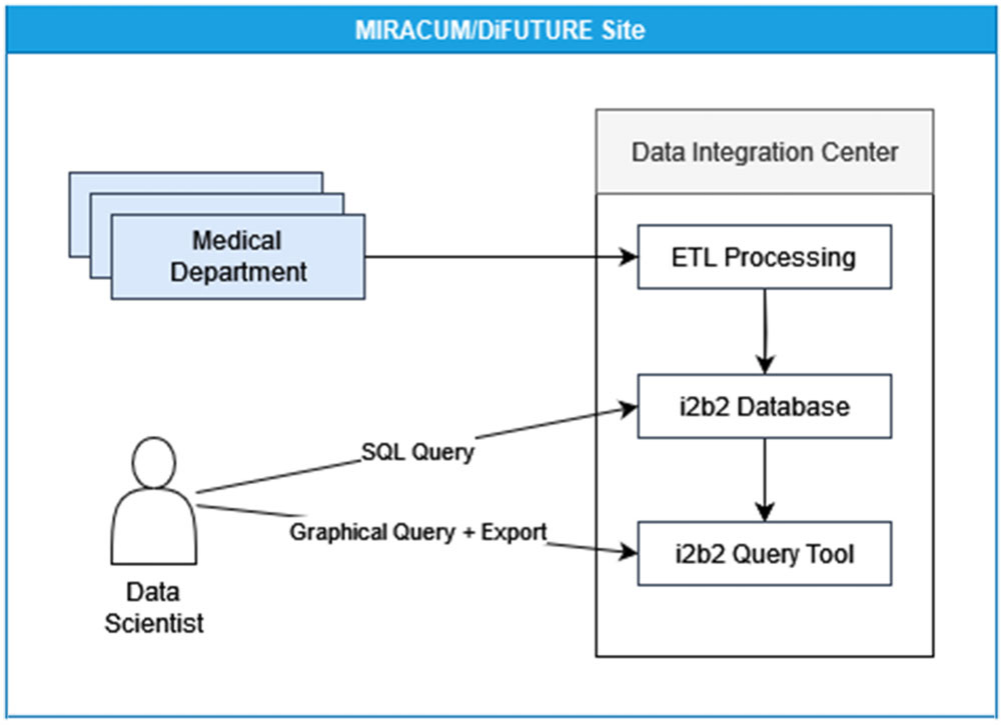
In MIRACUM and DIFUTURE every participating center establishes a DIC. At each site, multiple medical departments provide their clinical data to the DIC. The data is transformed and processed into an i2b2 database, while the i2b2 web application allows access to this data through the i2b2 web interface. Alternatively, direct database access is possible. Thus, any tool can be used to analyze data either based on exports or within a direct database connection.

The Medical Informatics Initiative^17^^,^^18^ funded by the German Federal Ministry of Education and Research aims to make data from patient care and research more usable. MIRACUM (Medical Informatics in Research and Care in University Medicine)^9^ and DIFUTURE (Data Integration for Future Medicine)^19^ are 2 of the consortia pursuing this goal by networking several German university hospitals, primarily through the use of open source software components. The first step of the data integration is to collect data from different systems of different departments in a hospital-internal data repository. For this purpose, each participating hospital has the task of setting up a so-called data integration center (DIC). It integrates data from different departments and different systems into a central i2b2 database, cf. Figure 2. The data from the different systems are first preprocessed and then transferred to the central system by specially developed extract, transform, and load (ETL) jobs. The database users with a background from different departments and scientific projects need appropriate access rights. Therefore, an environment was built to reflect these settings, including an evaluation dataset based on the i2b2 Harvard demo dataset.

Since the dataset is only 63 MB in size, we decided to enlarge it to simulate a more realistic environment. To the size of datasets, data of certain tables was replicated (*encounter_mapping*, *patient_mapping*, *visit_dimension*, *observation_fact*, and *patient_dimension*) by a certain size factor. For example, a factor of 2 would mean doubling the data. Similar to the configuration in a MIRACUM DIC, data separation was established. Four equally complex policies were defined that allowed data access to subsets of different sizes:

- Restricted access on data of provider ID *LCS-I2B2: D000109064*, which amounts ∼20% of overall data
- Restricted access on data of provider ID *LCS-I2B2: D000109061*, which amounts ∼10% of overall data
- Restricted access on data of provider ID *LCS-I2B2: D000109075*, which amounts ∼5% of overall data
- Restricted access on all remaining data, which amounts ∼65% of overall data

Finally, a total of 5 databases were created: a classic i2b2 data mart for each of the sets A–D, and one containing all the data and using security roles to implement access to the different sets.

### Evaluation

To measure the computational performance of our approach compared to the classical data mart, an evaluation was designed. Each DM was additionally implemented with RLS and attached to a specific user. These pairs of datasets from both approaches were then compared by checking the following condition: $\left|\left({\mathrm{RLS}}_{X\;}-\;X\right)\cup \left(X\;-\;{\mathrm{RLS}}_{X}\right)\right|=\;0\;\wedge \;\left|{\mathrm{RLS}}_{X}\right|=\;\left|X\right|$, where X is the respective set A–D and $\mathrm{RL}S_{X}$ indicates the respective implementation of set *X* using security roles. For each set, this condition was checked using a SQL script.

The subject of the performance evaluation were 3 queries of varying complexity. They were created via the i2b2 interface. The generated SQL code was extracted and executed outside the interface. Thus, the queries return the same result, such as executed inside the i2b2 interface. Informally, the queries were defined as follows:

1)  Get all female Patients with age 18–34.
2)  Select patients with blood count (HCT) values <50 entered by Kildare, Winchester, Kiley, Zorba, or Delgado.
3)  Find all patients who have ankle and foot or elbow and forearm injuries. Here, the data provider should be MD Trapper John McIntyre or MD Frank Burns.

These queries were applied to the 4 different datasets and both approaches. The number of results varied accordingly (cf. Table 1). In order to increase performance, *pg_prewarm* was enabled and 3 database indices were added to the existing i2b2 data schema: observation_fact(provider_id), observation_fact(patient_num, provider_id) and observation_fact(encounter_num, provider_id). For each dataset, approach, and query, 100 queries were run in random order to warm up the caches, and another 1000 queries were run randomly as a performance measure. After each benchmark run, the system was reset to flush the cache. In addition, the benchmark was run with datasets of different sizes increased by the resizing factors: $f\in \{2^{x}\;|\;x\in Z\wedge 5\leq x\leq 10\}$.

**Table 1.** For each query executed on a given subset, a different set of results is returned.

	Query 1	Query 2	Query 3
**A**	0	7	0
**B**	2	14	0
**C**	1	0	0
**D**	11	33	6

The table shows how many tuples the result sets include for a resizing factor of 1.

The performance is measured in transactions per second (TPS). The trend could be simply fitted with a hyperbolic function, $\mathrm{TPS}\;\mathrm{drop}\;\mathrm{per}\;\mathrm{MB}\left(s\right)=\;\frac{c}{s}$, where *s* is the data size and *c* is a rough estimate of the number of transactions per second, which usually decreases as the size of the database increases. Based on the TPS drop per MB for both approaches (RLS vs DM), we calculate the TPS ratio, that is, the TPS drop per MB for RLS divided by the TPS drop per MB for DM. Consequently, a TPS ratio less than one indicates that RLS was faster, while a TPS ratio greater than one indicates that DM was faster than RLS. A ratio of one is a performance tie.

**Figure 3.**
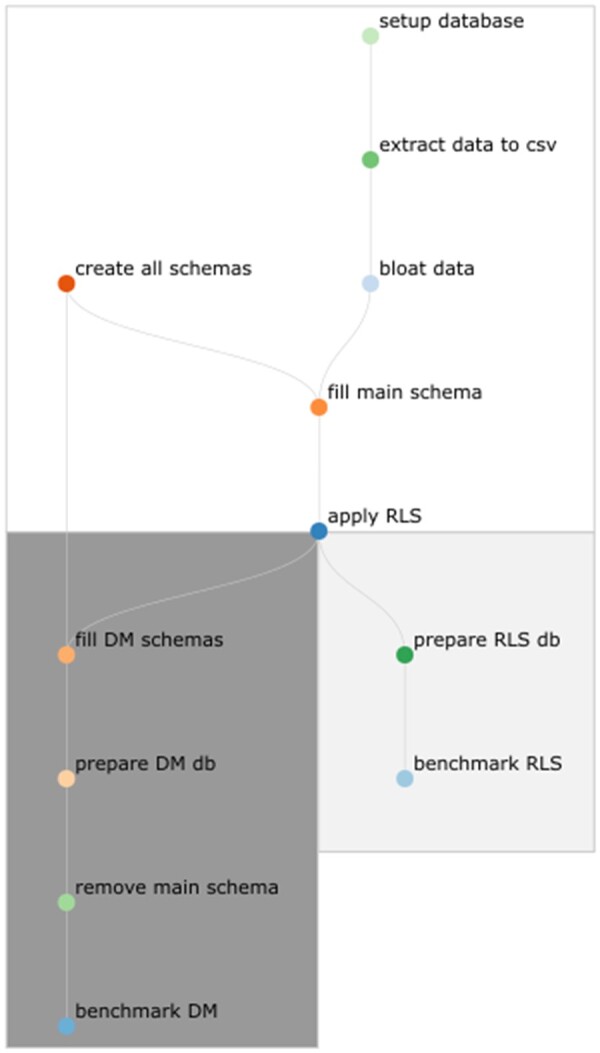
Flowchart of the benchmark process for both methods (read from top to bottom). Until the step “apply RLS”, the workflow is similar for both benchmarks. The main schema contains the entire dataset enriched with RLS policies. Starting from the database with these policies, the data mart (DM) schemas are created with little logical effort. To save storage space, the main schema is removed before benchmarking the data marts. No DMs are created when benchmarking RLS.

Just-in-time compilation of queries was globally disabled in PostgreSQL. The entire experiment pipeline was implemented using Snakemake (cf. Figure 3).^20^ The database host on which the benchmark was run has an Intel Core i7-8700 CPU @ 3.20GHz with 64GiB DDR4 RAM and uses an SSD for storage.

## Results

### Data segregation

In this work, data segregation is implemented by defining data marts using security roles based on RLS. Further, the i2b2 application is manipulated such that each data mart is modeled as a project. These projects are manageable as usual inside the i2b2 application and can be accessed via the user-interface as well as via direct database connection.

However, each data mart requires a special setup. First, the i2b2 application, including the specific database tables, must be manipulated, and second, the RLS must be validated and applied to the database. To orchestrate this process, we designed and implemented the CLI i2b2rls, which has been made publicly available in the Python Package Index (PyPi) and can therefore be downloaded using the package manager pip.

Instantiating a new i2b2 project requires a configuration in the Project Management (PM) Cell, a database schema containing the Data Repository, and a data source definition for the i2b2 Wildfly application server to access the new CRC.

**Figure 4.**
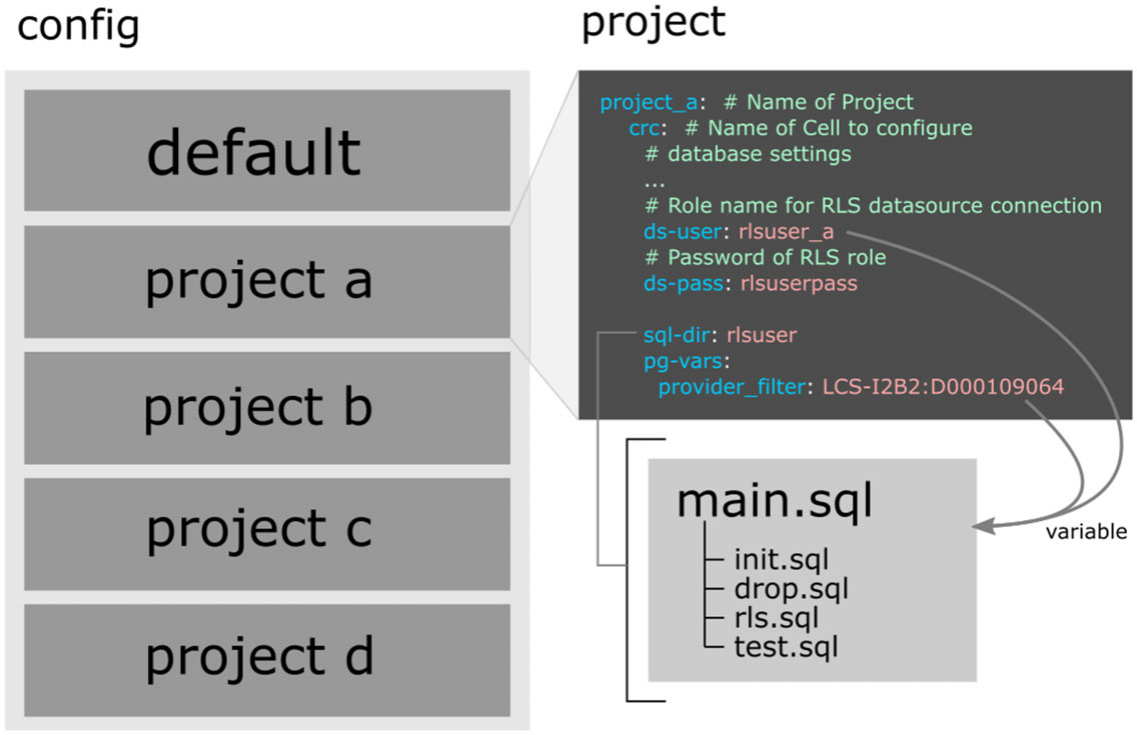
Exemplary representation of the configuration of the setup used in this case study. On the left is the structure of the configuration file with the default and 4 project sections. On the right is an excerpt from the project configuration that references the SQL files. An RLS SQL setup was sufficient, using the provider ID as a variable. The values are automatically transferred to SQL.

The CLI setup is designed to be defined in a central configuration file (yaml format) containing all project settings and several SQL files defining RLS policies and database settings. Figure 4 shows the required configuration for the use case of this study. In contrast to classic data marts, data is not replicated and therefore not kept redundant. Furthermore, everything is configured centrally using a main configuration file that contains both default settings and specific project definitions. In each project, the default settings can be overridden. In this way, RLS can be made generic and reused in multiple projects due to the possibility of user-defined variables. In our use case, RLS policies were written to separate data based on department numbers configured with such a variable. Several of these custom variables can be added and used in the required SQL code. Likewise, some configuration properties are automatically passed as variables. The main SQL file contains a rollback mechanism that only allows valid RLS definitions to be processed that have passed testing and did not trigger any other error. We propose a setup with 4 additional SQL files as default:

1)  init.sql: this file is intended for database initialization, such as module loading.
2)  drop.sql: in case the tests failed, certain resources might still exist. This file is executed to ensure a valid database after rollback.
3)  rls.sql: here the RLS policies are defined.
4)  test.sql: with the help of pgTAP test cases are defined here.

Beyond that, all features and extensions that Postgres offers can be integrated and used. The SQL code is computed using the native Postgres client, which offers several functions in addition to the actual database system. For each security role, a separate set of SQL files can be created in different folders. The number of files and the use of additional modules and structures can be changed. To use the full functionality, in-depth expertise on Postgres and knowledge of the i2b2 database is required. However, mastering these technologies provides extensive opportunities to realize the database setup with all the functionality of Postgres and its client, managing the entire configuration in a comfortable and compact way. Sample configurations are available on gitlab to support the use of the tool. In addition, due to the technical requirements of Postgres, we provide a Docker-based i2b2 stack to facilitate reproducibility and testing of our approach.

### Evaluation

**Figure 5.**
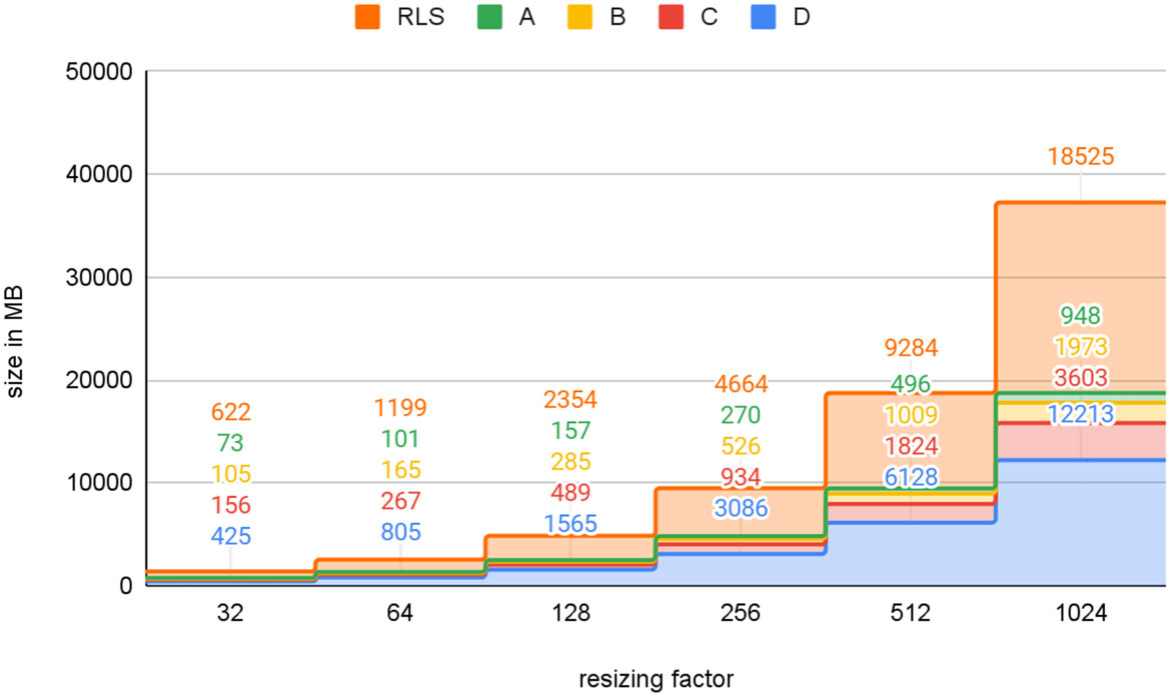
For the evaluation, 4 sets are defined and separated by the provider id. “RLS” is the database where the datasets are separated by policies. A–D form the corresponding data marts, which are created by resizing and replication.

In order to compare both data segregation approaches, namely DM and RLS, multiple datasets were prepared as described above. Figure 5 plots the size of these datasets. There are neglectably small discrepancies between the total size of classic data marts and the RLS set. For example, resizing factor 1024, the sum of all DMs, has 212 MB (∼1.14%) more data than the RLS dataset. The reason for that is the requirement of replication of some tables for each DM, which are not affected by the resizing process. More details about the datasets’ properties are provided in Supplementary Tables SA1 and SA2.

**Figure 6.**
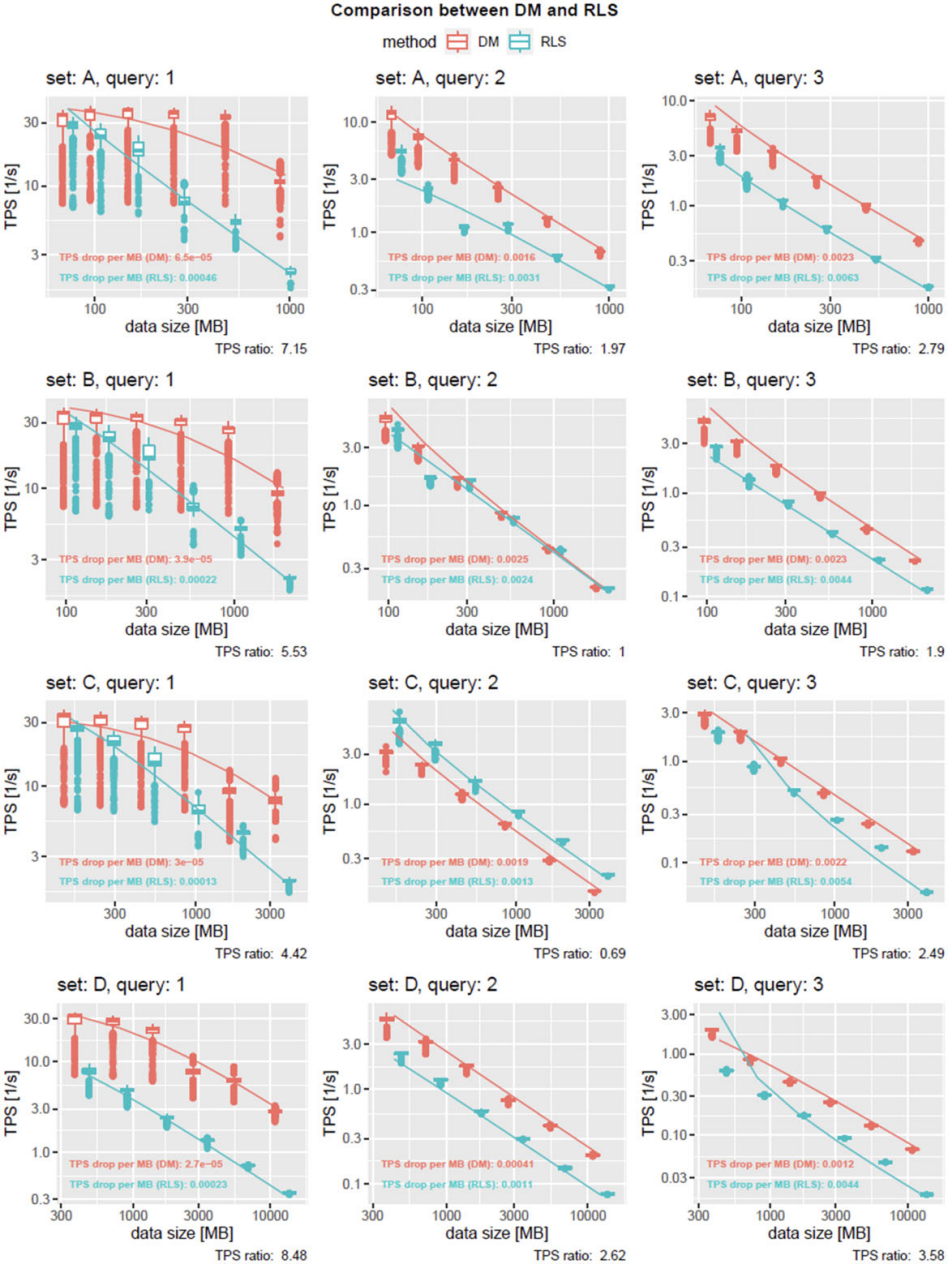
TPS dependency on the data size for various datasets and queries. Observed trend (box plots) and predicted trend (solid lines) are shown for DM (red) and RLS (blue). TPS drop (coefficient of the hyperbolic fit) is given for both the query methods, showing that RLS runs on average 3.55 and on median 2.71 times slower compared to DM. Note that the plots use logarithmic scaling.

For each measurement, that is, runs of queries on a certain dataset, multiple distributions were observed due to SQL query optimization and caching depending on which queries had been run previously. For this reason, significance tests were avoided for the experiments shown in Figure 6. Obviously, query 3 took the most computational demand for any constellation, followed by query 2 and 1. The latter one, interestingly, had the largest discrepancy between RLS and DM, visible in high TPS ratios ranging from 4.42 to 8.48 and in average x. Query 2 in average had the best TPS ratio of 1.57. It was the only query for which RLS outperformed DM (set C with query 2, returning 0 result rows) and the 2 approaches performed equally (set B with query 2, returning 14 result rows). Query 3 on average reached a TPS ratio of 2.69 while DM outperformed RLS in every constellation. However, in all other experiments, DM outperformed RLS. Based on the TPS ratios, DM is observed to be 3.55/2.71 times faster on average/median compared to RLS (cf. Supplementary Figure SA1). For average values in seconds, see Supplementary Table SC. We would like to strongly emphasize these values as general trends as obviously the performance of the approaches depends on the query, the dataset, and potentially the order of certain queries impacting the database’s cache. We assume that the more complex queries (2 and 3) already reduce the result set due to SQL joins, which most likely impacts the memory consumption and therefore the execution overhead of RLS. Nevertheless, although RLS is measured to be slower on average and median, data replication, and synchronization among projects is made obsolete. Notably, while defining RLS policies, one should be aware that they are computed per row. Therefore, query optimization should be considered. The more users and data, the more worthwhile the optimization effort. For the requirements of the use case described in this article, we already offer optimized code for the RLS policies. In cases of large data storage, the proposed approach might save a significant amount of data, but lead to noticeable performance drawbacks as shown in the experiments. For small datasets requiring many security roles, the approach might be attractive. In this case performance degradation might be not perceivable at all while development complexity can be drastically reduced as synchronization is not required and features such as role inheritance might leverage the implementation of new security roles as well.

## Discussion

The presented work introduces a new approach of data segregation for i2b2. It realizes project-specific data access rights using security roles based on RLS. Unlike the originally envisioned approach of i2b2, using DM, our approach does not require data replication and synchronization. We implemented our approach with a CLI using RLS, which is available for the latest Postgres databases and i2b2’s core features. Thus, no change of i2b2’s codebase was required and the approach can be applied to any i2b2 installation without the need for any additional software stack or heavy applications. Further, test-driven development and therefore security role validation were added to the framework to prevent errors in the case of sensitive access to medical records. From our point of view, errors could also happen while creating data marts in the classical way, especially when no test-driven development is used. There the data mart would be required to be entirely recreated as the data is replicated. In case of an error in a security role setup, it could be turned off instantly by disabling the affected database user. After adjusting the test cases and correcting its policy, it could be redeployed immediately. To authenticate users to the web client, i2b2 offers the option to query an LDAP server by setting the corresponding user parameters. The tedious task of manually setting these LDAP parameters was simplified by a feature implemented in the command line tool. Also, the Postgres server offers backend authentication through LDAP, which is reflected in an example configuration file in our Docker-based i2b2 stack. This leverages the possibility of a central user management concept as RLS also applies for direct database access.

Hodge and Milligan present a method for creating data marts using meta pointers for use by local software without the need to store the data locally.^21^ Similar to our work, their approach saves storage space and data transfer resources. However, to our knowledge, the implementation using RLS is not yet published in any related work. Further, we are the first in literature evaluating the performance of RLS in a data warehouse software. In our experiments, we experienced a drop of 3.55/2.71 TPS per MB on average/median.

Besides the impact on query performance, computational demand for replication, and the disk space usage itself is especially increased using the classical data mart approach. Shown by related works,^22–24^ synchronizing these data marts with the main database is a challenge that should not be underestimated. Finally, one ends up with a tradeoff decision between flexibility and implementation complexity at the price of query performance degradation and memory consumption. Therefore, our approach is especially useful in a scenario of many i2b2 projects. Due to role inheritance in Postgres, the presented approach might become less complex and more modularizable with an increasing number of RLS policies, to some extent comparable to database views.^25^ Therefore, we expect that over the time the effort of maintaining and creating security roles will decrease. Especially, compared to a manual data mart creation the proposed tool is advantageous. It could be used for project setup even solely, by deploying projects without RLS policies. How the deployment effort compares to an ETL-based implementation is not estimable. However, less complex ETL processes are likely and in some cases, it is advantageous to divide up complexities and responsibilities. Based on the presented approach, i2b2 was deployed as a clinical data storage solution at the MIRACUM site in Freiburg.

Finally, although our implementation relies on Postgres, there are other DBMSes that also support RLS, such as Microsoft SQL Server (MSSQL), ORACLE Database and ClickHouse. Therefore, the presented approach can be considered as a general pattern for data segregation.

Necessary staff expertise and higher resources for implementation of the RLS i2b2 approach may limit its applicability at many sites. However, the publication of our code as a skeleton, might decrease the learning curve and Postgres as well as i2b2 are open source projects with an active and helpful community, which facilitates learning, too.

The case study presented, only provides benchmark results of a single implementation and cannot be generalized. The experiment was limited to only 3 queries and performed on generated datasets. Databases are complex systems with complicated caching functionality, which would behave differently in a real scenario. Also, we did not deeper investigate database optimization techniques such as partitioning. The used datasets were generated using replication, based on the Harvard demo dataset. Hence, results of this case study are limited. We are aware that this may affect the caching of the database in an unnatural way and may not necessarily resemble the behavior of a real database in terms of computation. However, memory consumption, which is a critical issue for a database, can be simulated in this way. Therefore, the results are certainly not real, but they give direction and help to make an estimate. The present data clearly indicate that performance loss due to RLS is tolerable and are outweighed by benefits in functionality and security.

In future works, query performance of RLS might be further investigated. On the one hand, the generated SQL queries by i2b2, the database model, and the RLS rules could be further optimized. Also a case study applied on a different and more realistic dataset could give better performance insights. Finally, the approach could be implemented for other DBMSes that are compatible with i2b2, such as ORACLE and MSSQL. This would require extending the AutoMapDB framework^16^ to include these 2 systems, extending the i2b2rls tool to use the respective database CLI client, and writing RLS for the respective DBMS. However, unlike for ORACLE, there is no TAP implementation for MSSQL. This would still need to be implemented or solved by a comparable library. Extending the DBMS compatibility would also allow a performance comparison of the approach among the supported systems.

## Conclusion

We presented an approach to realize data segregation in one i2b2 database without the need for data replication and synchronization. The approach was realized by security roles and with the help of RLS, which is a standard feature of recent PostgreSQL versions. We succeeded in developing an approach that can be applied to existing i2b2 systems without a modification of the core code. The proposed approach offers flexibility in defining security roles and seamlessly integrates with any i2b2 installation through a CLI tool that conveniently applies necessary changes into the i2b2 application. Similar to Wagholikar et al,^26^ a dockerized i2b2 stack is offered, which contains all required functionalities. Additionally, to the CLI, we published sample configuration used in this work. This allows the approach to be implemented with minimal migration and enables rapid implementation of custom security roles. As shown in our experiments, the approach on average is 3.55 and on median 2.71 times slower compared to classic i2b2 data marts potentially requiring data replication and synchronization. Security roles can be used for both constraining access via the i2b2 interface as well as constraining direct database access. Finally, we hope that the presented approach will help to simplify existing i2b2 infrastructures using data marts and provide a lightweight approach for the realization of data segregation in emerging i2b2 deployments.

## Supplementary Material

ooad068_Supplementary_Data

## Data Availability

All software and the base data set used in this work, including the i2b2rls CLI, sample configurations, code for dataset enlargement and the evaluation, and the i2b2 Docker stack, are published under open source licenses at https://gitlab.com/mds-imbi-freiburg/i2b2.The i2b2rls tool can be installed with the python package manager, pip, and is also available at https://pypi.org/project/i2b2rls.

## References

[1] Abouelmehdi K , Beni-Hessane A, Khaloufi H. Big healthcare data: preserving security and privacy. J Big Data. 2018;5(1). 10.1186/s40537-017-0110-7

[2] Househ M , Aldosari B, Alanazi A, et al Big data, big problems: a healthcare perspective. Stud Health Technol Inform. 2017;238:36-39. 28679881

[3] Shahid A , Nguyen T-AN, Kechadi M-T. Big data warehouse for healthcare-sensitive data applications. Sensors. 2021;21(7):2353. 10.3390/s21072353 33800574 PMC8037603

[4] Johnson SB , Paul T, Khenina A. Generic database design for patient management information. In: *Proc AMIA Annu Fall Symp*. Nashville, TN: American Medical Informatics Association; 1997:22–26. PMC2233478 9357581

[5] Nadkarni PM , Brandt C. Data extraction and ad hoc query of an entity—attribute—value database. J Am Med Inform Assoc JAMIA. 1998;5(6):511-527. 9824799 10.1136/jamia.1998.0050511 PMC61332

[6] Hong N , Li Z, Kiefer RC, et alBuilding an i2b2-based integrated data repository for cancer research: a case study of ovarian cancer registry. In: Wang F, Yao L, Luo G, eds. Data Management and Analytics for Medicine and Healthcare. Cham: Springer International Publishing; 2017:121-135. 10.1007/978-3-319-57741-8_8

[7] Ganslandt T , Mate S, Helbing K, et al Unlocking data for clinical Research—the German i2b2 experience. Appl Clin Inform. 2011;2(1):116-127. 10.4338/ACI-2010-09-CR-0051 23616864 PMC3631913

[8] Klann JG , Phillips LC, Herrick C, et al Web services for data warehouses: OMOP and PCORnet on i2b2. J Am Med Inform Assoc. 2018;25(10):1331-1338. 10.1093/jamia/ocy093 30085008 PMC6188504

[9] Prokosch H-U , Acker T, Bernarding J, et al MIRACUM: medical informatics in research and care in university medicine. Methods Inf Med. 2018;57(S 01):e82-e91. 10.3414/ME17-02-0025 30016814 PMC6178200

[10] PostgreSQL: Documentation: 9.5: Row Security Policies. Accessed December 13, 2019. https://www.postgresql.org/docs/9.5/ddl-rowsecurity.html

[11] PostgreSQL: Documentation: 9.5: Privileges. Accessed December 13, 2019. https://www.postgresql.org/docs/9.5/ddl-priv.html

[12] Improve RLS Planning by Marking Individual Quals With Security Levels. postgres/postgres@215b43c. GitHub. Accessed December 13, 2019. https://github.com/postgres/postgres/commit/215b43cdc8d6b4a1700886a39df1ee735cb0274d

[13] relationship Between Several i2b2 Components for New Projects. Accessed February 10, 2021. https://groups.google.com/g/i2b2-install-help/c/nYI3T16IfQk?pli=1

[14] Home—Test Anything Protocol. Accessed December 16, 2019. http://testanything.org/

[15] pgTAP: Unit Testing for PostgreSQL. Accessed December 11, 2019. https://pgtap.org/

[16] Thomczyk F , Scheible R. AutoMapDB: Automagically Mapping of Postgres Database Schema into a Python Abstraction. 2021. 10.5281/zenodo.5569138

[17] Semler SC , Wissing F, Heyder R. German medical informatics initiative. Methods Inf Med. 2018;57(S 01):e50-e56. 10.3414/ME18-03-0003 30016818 PMC6178199

[18] Gehring S , Eulenfeld R. German medical informatics initiative: unlocking data for research and health care. Methods Inf Med. 2018;57(S 01):e46-e49. 10.3414/ME18-13-0001 30016817 PMC6178201

[19] Prasser F , Kohlbacher O, Mansmann U, et al Data integration for future medicine (DIFUTURE). Methods Inf Med. 2018;57(S 01):e57-e65. 10.3414/ME17-02-0022 30016812 PMC6178202

[20] Mölder F , Jablonski KP, Letcher B, et al Sustainable data analysis with Snakemake. 2021. 10.12688/f1000research.29032.1 PMC8114187 34035898

[21] Hodge LK , Milligan M. Managing virtual data marts with metapointer tables. In: *Proceedings of the 36th Annual Hawaii International Conference on System Sciences, 2003.* Big Island, HI: IEEE; 2003:7 pp. 10.1109/HICSS.2003.1174606

[22] Ying-Chun S , Qiu-Ju Z, Jing L. A Kind of heterogeneous database synchronization mechanism. In: *2016 International Conference on Intelligent Transportation, Big Data Smart City (ICITBS)*. 2016:168–171. 10.1109/ICITBS.2016.89

[23] Scriney M , McCarthy S, McCarren A, et al Automating data mart construction from semi-structured data sources. Comput J. 2019;62(3):394-413. 10.1093/comjnl/bxy064

[24] H, Krasniqi Z , Ahmeti E, Gjonaj A. The methodology of data collecting and the real time synchronization in ETL. Int J Comput Sci Inf Secur. 2017;15(11):116-122.

[25] Roussopoulos N. Materialized views and data warehouses. ACM SIGMOD Rec. 1998;27(1):21-26. 10.1145/273244.273253

[26] Wagholikar KB , Dessai P, Sanz J, et al Implementation of informatics for integrating biology and the bedside (i2b2) platform as docker containers. BMC Med Inform Decis Mak. 2018;18(1):66. 10.1186/s12911-018-0646-2 30012140 PMC6048900

