# Supplementary material for: Integrating row level security in i2b2: segregation of medical records into data marts without data replication and synchronization
Source: JAMIA Open. 2023 Aug 14;6(3):ooad068. doi: 10.1093/jamiaopen/ooad068 (PMC10425194; doi:10.1093/jamiaopen/ooad068)
Supplement: ooad068_Supplementary_Data [file ooad068_supplementary_data.docx]

# Appendix

| **Table / f** | **1** | **32** | **64** | **128** | **256** | **512** | **1024** |
| --- | --- | --- | --- | --- | --- | --- | --- |
| **code_lookup** | 350 | 350 | 350 | 350 | 350 | 350 | 350 |
| **concept_dimension** | 190660 | 190660 | 190660 | 190660 | 190660 | 190660 | 190660 |
| **encounter_mapping** | 12386 | 396352 | 792704 | 1585408 | 3170816 | 6341632 | 12683257 |
| **modifier_dimension** | 75 | 75 | 75 | 75 | 75 | 75 | 75 |
| **observation_fact** | 90853 | 2906099 | 5813395 | 11626578 | 23259736 | 46523736 | 93050361 |
| **patient_dimension** | 134 | 4288 | 8576 | 17152 | 34304 | 68608 | 137216 |
| **patient_mapping** | 3650 | 116800 | 233600 | 467200 | 934400 | 1868800 | 3737600 |
| **provider_dimension** | 62 | 62 | 62 | 62 | 62 | 62 | 62 |
| **visit_dimension** | 12387 | 396384 | 792768 | 1585536 | 3171072 | 6342144 | 12684286 |

***Table A1:*** *Number of rows of the particular table given different resizing factors (f).*

| **f** | **RLS** | **A** | **B** | **C** | **D** |
| --- | --- | --- | --- | --- | --- |
| 1 | 62 MB | 45 MB | 46 MB | 48 MB | 56 MB |
| 2 | 80 MB | 46 MB | 48 MB | 51 MB | 68 MB |
| 4 | 116 MB | 48 MB | 52 MB | 58 MB | 92 MB |
| 8 | 189 MB | 52 MB | 60 MB | 72 MB | 140 MB |
| 16 | 333 MB | 59 MB | 75 MB | 100 MB | 235 MB |
| 32 | 622 MB | 73 MB | 105 MB | 156 MB | 425 MB |
| 64 | 1199 MB | 101 MB | 165 MB | 267 MB | 805 MB |
| 128 | 2354 MB | 157 MB | 285 MB | 489 MB | 1565 MB |
| 256 | 4664 MB | 270 MB | 526 MB | 934 MB | 3086 MB |
| 512 | 9284 MB | 496 MB | 1009 MB | 1824 MB | 6128 MB |
| 1024 | 18525 MB | 948 MB | 1973 MB | 3603 MB | 12213 MB |

***Table A2:*** *Size in MB of the different sets accessed by security roles (A-D) and the entire database containing the full dataset (RLS) given different resizing factors (f).*

|  | resizing factor | 1 | | 2 | | 4 | | 8 | | 16 | | 32 | | 64 | | 128 | | 256 | | 512 | | 1024 | |
| --- | --- | --- | --- | --- | --- | --- | --- | --- | --- | --- | --- | --- | --- | --- | --- | --- | --- | --- | --- | --- | --- | --- | --- |
|  | approach | rls | dm | rls | dm | rls | dm | rls | dm | rls | dm | rls | dm | rls | dm | rls | dm | rls | dm | rls | dm | rls | dm |
| set | query |  |  |  |  |  |  |  |  |  |  |  |  |  |  |  |  |  |  |  |  |  |  |
| A | 1 | 63.51 | 61.0 | 59.57 | 64.21 | 54.09 | 55.37 | 49.69 | 47.3 | 40.03 | 40.92 | 38.6 | 36.81 | 44.56 | 32.82 | 54.7 | 32.22 | 129.71 | 32.48 | 188.49 | 33.26 | 445.69 | 94.09 |
|  | 2 | 71.59 | 65.69 | 75.92 | 69.44 | 87.09 | 69.57 | 77.64 | 67.15 | 107.07 | 76.87 | 189.22 | 93.09 | 402.94 | 141.93 | 894.57 | 227.7 | 843.99 | 398.9 | 1618.0 | 745.7 | 3094.06 | 1495.15 |
|  | 3 | 130.7 | 125.65 | 129.94 | 130.92 | 127.64 | 128.62 | 134.37 | 126.25 | 161.02 | 128.85 | 282.02 | 148.89 | 552.59 | 198.23 | 881.1 | 306.79 | 1580.64 | 532.53 | 3121.15 | 981.18 | 5822.99 | 2134.3 |
| B | 1 | 64.19 | 66.61 | 57.85 | 64.27 | 53.3 | 55.46 | 49.65 | 47.08 | 40.75 | 41.44 | 38.9 | 34.68 | 46.19 | 33.87 | 58.08 | 34.59 | 135.15 | 36.78 | 197.74 | 41.73 | 448.01 | 110.54 |
|  | 2 | 78.3 | 77.66 | 97.05 | 84.75 | 80.13 | 92.53 | 96.63 | 126.02 | 149.94 | 138.38 | 241.2 | 199.74 | 597.97 | 331.21 | 625.08 | 606.55 | 1261.5 | 1162.14 | 2363.12 | 2280.31 | 4902.41 | 4788.36 |
|  | 3 | 131.74 | 135.44 | 132.79 | 140.23 | 137.26 | 137.57 | 151.67 | 141.99 | 219.49 | 159.84 | 361.51 | 210.69 | 743.72 | 324.29 | 1220.18 | 555.14 | 2390.15 | 1021.44 | 4382.54 | 2219.87 | 8574.07 | 4466.97 |
| C | 1 | 63.93 | 66.04 | 59.63 | 63.49 | 54.16 | 56.45 | 49.09 | 47.19 | 42.63 | 41.73 | 40.76 | 36.75 | 50.06 | 35.9 | 66.46 | 37.47 | 148.27 | 41.75 | 221.9 | 110.78 | 500.74 | 127.29 |
|  | 2 | 72.61 | 78.47 | 72.21 | 84.01 | 75.22 | 99.73 | 90.13 | 123.3 | 110.69 | 162.82 | 163.66 | 323.64 | 270.55 | 423.36 | 600.12 | 808.53 | 1181.72 | 1537.84 | 2192.68 | 3441.56 | 4768.43 | 6814.62 |
|  | 3 | 138.41 | 138.53 | 143.39 | 141.03 | 155.82 | 150.67 | 186.7 | 167.51 | 311.69 | 213.73 | 527.13 | 350.58 | 1131.55 | 520.17 | 1947.5 | 949.99 | 3731.66 | 2050.98 | 7061.05 | 4120.85 | 19527.26 | 7715.46 |
| D | 1 | 71.5 | 68.07 | 68.34 | 63.22 | 73.91 | 58.33 | 79.19 | 50.5 | 92.1 | 43.45 | 131.49 | 39.31 | 210.27 | 40.09 | 416.8 | 48.97 | 734.52 | 130.82 | 1394.92 | 161.86 | 2839.04 | 357.29 |
|  | 2 | 95.09 | 77.96 | 103.5 | 86.34 | 130.45 | 91.21 | 172.05 | 104.13 | 249.6 | 132.18 | 424.43 | 186.74 | 805.92 | 320.7 | 1695.83 | 573.2 | 3318.04 | 1322.32 | 6819.46 | 2438.6 | 12940.74 | 4982.64 |
|  | 3 | 155.63 | 147.39 | 178.53 | 155.54 | 215.71 | 179.75 | 309.3 | 225.57 | 708.24 | 319.45 | 1607.6 | 523.08 | 3210.55 | 1170.26 | 5669.09 | 2191.66 | 10824.35 | 3990.86 | 21413.77 | 7603.56 | 53839.3 | 15078.5 |

***Table A3****: Showing the computation time in ms of the transactions in average for the respective query, set and approach. Overall the average is 779.83 ms for DM vs 1730.61 ms for RLS per transaction.*

| 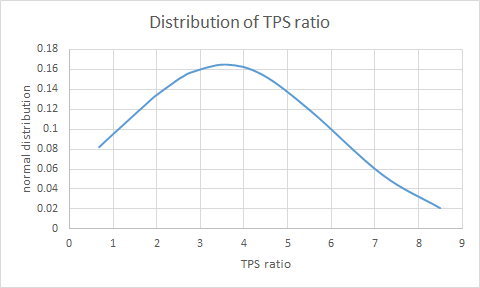  (a) | 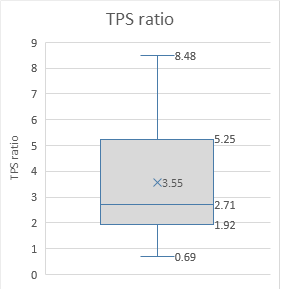  (b) |
| --- | --- |

***Figure A1:*** *Distribution (a) and boxplot (b) of the TPS ratio.*
